# Exploration of iodine adsorption performance of pyrene-based two-dimensional covalent organic frameworks†

**DOI:** 10.1039/d4ra04994b

**Published:** 2024-08-15

**Authors:** Weican He, Shenglin Wang, Hui Hu, Jiaxin Yang, Tiao Huang, Xiaofang Su, Songtao Xiao, Jianyi Wang, Yanan Gao

**Affiliations:** a Key Laboratory of Ministry of Education for Advanced Materials in Tropical Island Resources, Hainan University No 58, Renmin Avenue Haikou 570228 China 21220856000126@hainanu.edu.cn ygao@hainanu.edu.cn; b China Institute of Atomic Energy Beijing 102413 China xiao200112@163.com

## Abstract

Radioiodine (mainly ^129^I and ^131^I) is known to be dangerous nuclear waste due to its high toxicity, fast mobility and long radioactive half-life. As an emerging class of novel porous organic polymers, covalent organic frameworks (COFs) have demonstrated tremendous application potential in the field of radioactive iodine capture because of their high specific surface area and tunable pore structure. Herein, three π-conjugated pyrene-based COFs, namely PyTTA-BPDA-COF, PyTTA-BPY-COF, and PyTTA-BT-COF, have been successfully prepared and used as highly efficient adsorbents for iodine capture. The experimental results show that the three COFs displayed excellent adsorption performance, with adsorption capacity of 5.03, 4.46, and 3.97 g g^−1^ for PyTTA-BPDA-COF, PyTTA-BPY-COF, and PyTTA-BT-COF, respectively. Additionally, the release rate of iodine-loaded COFs in methanol solution and recyclability were also impressive, demonstrating their potential for practical applications. The mechanism investigation reveals that both imine linkage and π-conjugated structure of the COFs may contribute to their high iodine adsorption capability. This work is instructive as a guide for designing and synthesizing COFs as a solid-phase adsorbent for iodine uptake.

## Abstract 2

Radioiodine (mainly ^129^I and ^131^I) is known to be dangerous nuclear waste due to its high toxicity, fast mobility and long radioactive half-life.

## Graphical abstract

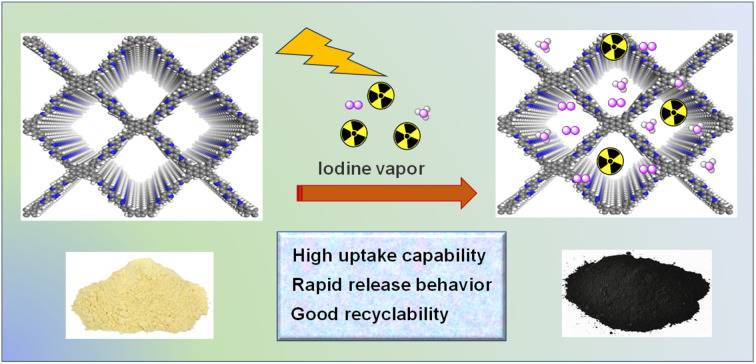

## Introduction

With the rapid pace of industrialization and modernization, environmental pollution and energy shortages have emerged as pressing issues, requiring the urgent development of efficient and environmentally friendly materials and technologies.^1–6^ With the depletion of fossil fuels and the intensification of the greenhouse effect, nuclear energy has garnered increased attention in recent years.^7^ Nevertheless, radionuclides would be released into the environment during the entire process of nuclear power generation. In the fields of environmental remediation and energy storage, radioactive iodine (^129^I and ^131^I) is a common pollutant associated with the expansion of nuclear power generation. Due to its high chemical activity and long half-life (such as 1.6 × 10^7^ years for ^129^I), it poses significant risks to both human health and the environment.^8,9^ Therefore, iodine adsorption technology has garnered widespread attention due to the unique application value.^10^ However, the limitations of traditional iodine adsorption materials in adsorption capacity, selectivity, and stability hinder them from meeting the increasingly stringent environmental and energy demands. The structural properties of the adsorbent (such as surface area, pore size, and pore volume), the affinity of the binding site to the I_2_ molecule, and the density of the binding site all play a crucial role in determining adsorption capacity. Achieving a balance between these factors is essential for maximizing adsorption capacity of adsorbent materials. Consequently, the development of novel and efficient iodine adsorption materials has become a hotspot of current research.

Covalent organic frameworks (COFs), as an emerging class of porous organic materials characterized by high specific surface area, tunable pore size, and excellent thermal and chemical stability, hold significant promise across various fields, such as gas adsorption, separation, and catalysis.^11–13^ Notably, COFs incorporated with extended π-conjugated structure can provide new avenues for the design of efficient iodine adsorption materials due to their specific electronic structure and chemical properties.^14–16^ The introduction of π-conjugation structure can not only adjust the physical and chemical properties of COFs but also enhance the interaction with iodine molecules, thus improving adsorption efficiency and selectivity.^17–21^ Pyrene is a well-known excellent electron donor. Its large π-conjugated structure ensures rapid electron-transfer abilities, which is favorable for the formation of charge transfer complexes between the iodine molecules and pyrene moiety of adsorbents.

Therefore, we designed and synthesized a series of pyrene-containing COFs to systematically investigate their iodine adsorption performance and mechanism, providing theoretical insights and experimental foundations for the development of novel and efficient iodine adsorption materials. Specifically, this study focused on the preparation of three pyrene-based two-dimensional (2D) COFs and evaluated their iodine adsorption performance through iodine vapor adsorption experiments. Additionally, it optimized their iodine adsorption performance by adjusting parameters such as pore size, surface properties, and chemical functional groups of COFs to meet the requirements of practical applications. This study deepens the understanding of the performance characteristics and application potential of extended π-conjugation COFs in the field of iodine adsorption, throwing insights into the design of novel and efficient iodine adsorption materials. Moreover, this research can serve as a valuable reference for exploring the application of COFs in response to challenges of energy and environment.

## Experimental section

### Synthesis of PyTTA-BPDA-COF

**Scheme 1:**
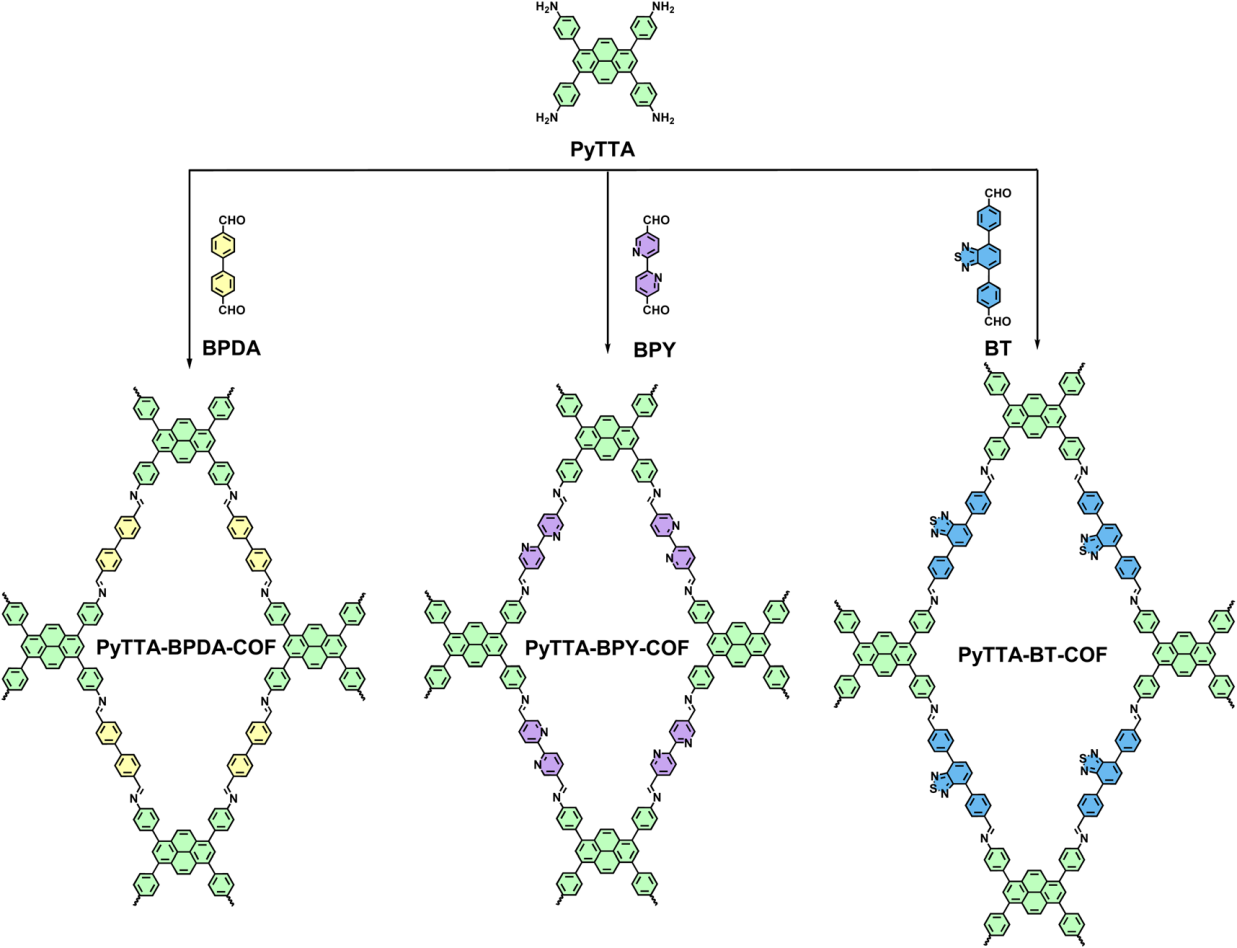
Synthetic route of PyTTA-BPDA-COF, PyTTA-BPY-COF and PyTTA-BT-COF.

PyTTA-BPDA-COF was synthesized according to the reported literature^22^ (Scheme 1). Firstly, PyTTA (21.0 mg, 37 μmol) and BPDA (12.6 mg, 60 μmol) were dispersed in a mixture of *o*-xylene/BuOH (667 μL/333 μL) in a 10 mL Pyrex tube through sonication for 15 minutes. Then, 0.1 mL acetic acid (6 M) was added to the tube and sonicated for another 2 minutes. Next, the tube was rapidly frozen in a liquid nitrogen bath at 77 K and subjected to three cycles of freeze-thaw with a cryogenic pump for evacuation. Afterward, the tube was flame-sealed and heated at 120 °C for 3 days. Upon cooling to room temperature, the precipitate was collected by filtration and washed separately with dry DMF and THF. Finally, the powder was vacuum-dried overnight at 120 °C, resulting in yellow PyTTA-BPDA-COF with a yield of 85%.

### Synthesis of PyTTA-BPY-COF

PyTTA-BPY-COF was synthesized according to the reported literature^23^ (Scheme 1). Specifically, PyTTA (22.6 mg, 40 μmol) and BPY (17 mg, 80 μmol) were dispersed in a mixture of *o*-DCB/BnOH (0.5 mL/0.5 mL) in a 10 mL Pyrex tube by sonication for 15 minutes. After adding acetic acid (6 M, 0.1 mL), the mixture was sonicated for another 2 minutes. Then, the tube was rapidly frozen in a liquid nitrogen bath at 77 K and subjected to three cycles of freeze-thaw to evacuate. Next, the tube was flame-sealed and heated at 120 °C for 3 days. After cooling to room temperature, the precipitate was collected by filtration and washed separately with dry DMF and THF. Finally, the powder was vacuum-dried overnight at 120 °C to yield brown PyTTA-BPY-COF with a yield of 85%.

### Synthesis of PyTTA-BT-COF

The experimental procedures in the literature^24^ were adopted in PyTTA-BT-COF synthesis (Scheme 1). Specifically, PyTTA (11.3 mg, 20 μmol) and BT (13.7 mg, 40 μmol) were dispersed in a mixture of *o*-DCB/BuOH (0.5 mL/0.5 mL) in a 10 mL Pyrex tube by sonication for 15 minutes. Then, the tube was added by acetic acid (6 M, 0.1 mL) and subjected to sonication for another 2 minutes. Next, it was rapidly frozen in a liquid nitrogen bath at 77 K and underwent three cycles of freeze-thaw to evacuate. Subsequently, the tube was flame-sealed and heated at 120 °C for 3 days. After cooling to room temperature, the precipitate was collected by filtration and washed with dry DMF and THF, respectively. The powder was vacuum-dried overnight at 120 °C to obtain orange-red PyTTA-BT-COF with a yield of 88%.

## Results and discussion

### Characterization of COFs

**Fig. 1:**
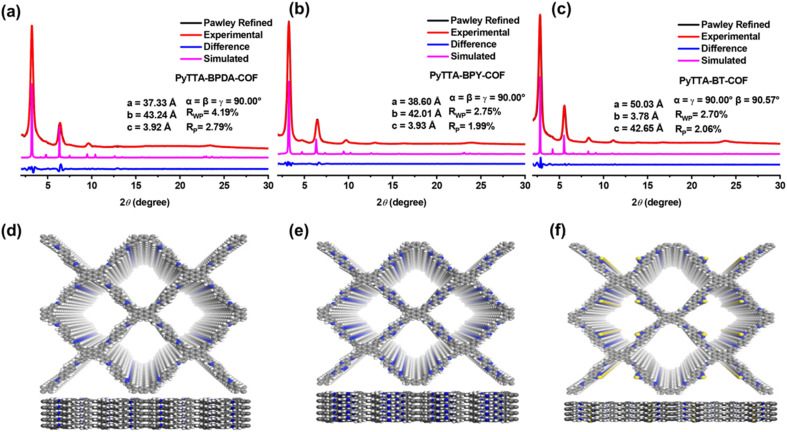
PXRD profiles of PyTTA-BPDA-COF (a), PyTTA-BPY-COF (b), PyTTA-BT-COF (c) for experimentally observed, Pawley refined, calculated for the AA stacking model, and the difference between experimental and calculated data.

As shown in Fig. 1a, the X-ray diffraction peaks indicate that PyTTA-BPDA-COF possesses a long-range ordered crystalline structure. The lattice parameters of PyTTA-BPDA-COF include *a* = 37.53 Å, *b* = 43.64 Å, *c* = 3.42 Å, *α* = *β* = *γ* = 90°. After refinement, these values are *a* = 37.33 Å, *b* = 43.24 Å, *c* = 3.92 Å, *α* = *β* = *γ* = 90°. The refinement coefficients *R*_p_ and *R*_wp_ are 2.79% and 4.19%, respectively. The refinement results imply that the PXRD curve derived from the AA stacking model of PyTTA-BPDA-COF closely matches the experimentally measured PXRD curve. Fig. 1b shows the lattice parameters of PyTTA-BPDA-COF: *a* = 38.84 Å, *b* = 41.81 Å, *c* = 3.63 Å, *α* = *β* = *γ* = 90°. After refinement, these parameters are *a* = 38.60 Å, *b* = 42.01 Å, *c* = 3.93 Å, *α* = *β* = *γ* = 90°. The refinement coefficients *R*_p_ and *R*_wp_ are 1.99% and 2.75%, respectively. The refinement results verify high conformity between the PXRD curve simulated by the AA stacking model of PyTTA-BPY-COF and that obtained from the experiment. According to Fig. 1c, the lattice parameters of PyTTA-BT-COF are *a* = 50.23 Å, *b* = 3.46 Å, *c* = 42.85 Å, *α* = *γ* = 90°, *β* = 90.57°. Upon refinement, these values are *a* = 50.03 Å, *b* = 3.76 Å, *c* = 42.65 Å, *α* = *γ* = 90°, *β* = 90.57°. The refinement coefficients *R*_p_ and *R*_wp_ are 2.06% and 2.70%, respectively. The refinement results indicate that the simulated PXRD curve of the AA stacking model of PyTTA-BT-COF aligns with the experimentally measured PXRD curve. The simulated crystal structures are shown in Fig. 1d–f.

**Fig. 2:**
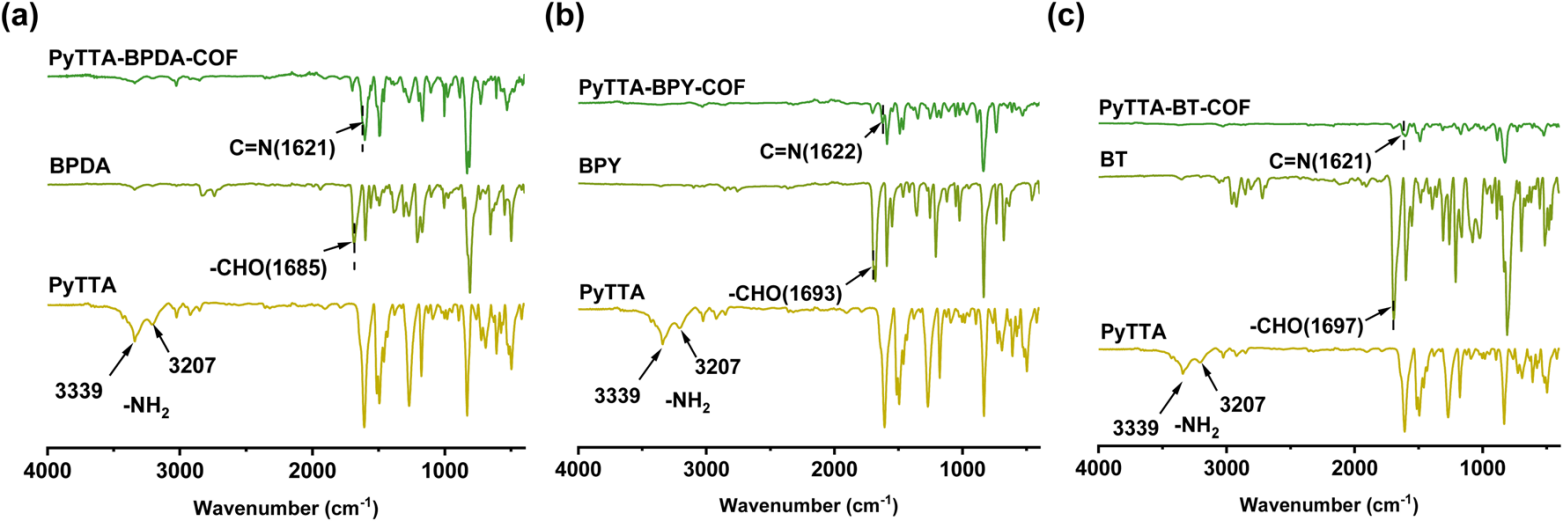
FT-IR spectra of BPDA, PyTTA and PyTTA-BPDA-COF (a); BPY, PyTTA and PyTTA-BPY-COF (b); BT, PyTTA and PyTTA-BT-COF (c).

FT-IR spectra provide abundant information about the molecular structure and functional groups of COFs. In the infrared spectra, vibration peaks correspond to specific chemical bonds or functional groups. Fig. 2 illustrates that the stretching vibrations of the –NH_2_ group of PyTTA monomer appear at 3207 cm^−1^ and 3339 cm^−1^. After the formation of PyTTA-BPDA-COF, PyTTA-BPY-COF, and PyTTA-BT-COF, the characteristic peak of the –NH_2_ group was remarkably weakened. Similarly, the characteristic peaks of the –CHO group of BPDA, BPY, and BT (appeared at 1685 cm^−1^, 1693 cm^−1^, and 1697 cm^−1^, respectively) display a noticeable diminish in stretching vibrations after COF synthesis. The generation of new characteristic peak of the C=N bond (at 1621 or 1622 cm^−1^) in all three COFs further confirms the successful condensation of COFs.^25^

**Fig. 3:**
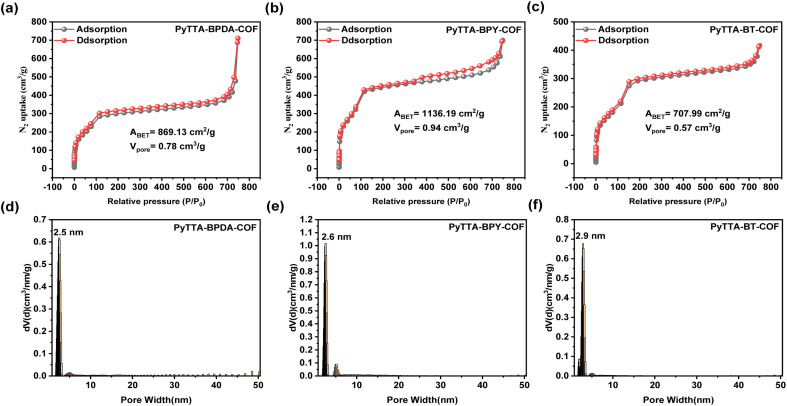
N_2_ adsorption isotherms and pore size distribution of PyTTA-BPDA-COF (a and d), PyTTA-BPY-COF (b and e), and PyTTA-BT-COF (c and f).

To investigate the pore structure of PyTTA-BPDA-COF, PyTTA-BPY-COF, and PyTTA-BT-COF, isothermal nitrogen adsorption–desorption measurements were conducted at 77 K. The results present typical IV-type isotherms in all three COFs (Fig. 3a–c), indicating the mesoporous nature of the three COFs.^26^ Based on the BET analysis, the specific surface areas of PyTTA-BPDA-COF, PyTTA-BPY-COF, and PyTTA-BT-COF are 869.13, 1136.19, and 707.99 m^2^ g^−1^, respectively, and the pore volumes are 0.78, 0.94, and 0.57 cm^3^ g^−1^, respectively. Subsequently, the density functional theory (DFT) was adopted to calculate the average pore diameters of the three COFs, which are 2.5 nm, 2.6 nm, and 2.9 nm, respectively (Fig. 3d–f), close to the theoretical values. Therefore, these three materials exhibit significantly high specific surface areas and pore volumes. High pore volumes provide more space to accommodate more guest molecules or ions, enhancing the adsorption and storage capacities of the materials. Moreover, all three materials demonstrate a well-defined mesoporous structure. The textural properties of the COFs, involving specific surface, pore size and pore volume we well as the iodine capture capability, are listed in Table S1.†

### Iodine vapor adsorption

**Fig. 4:**
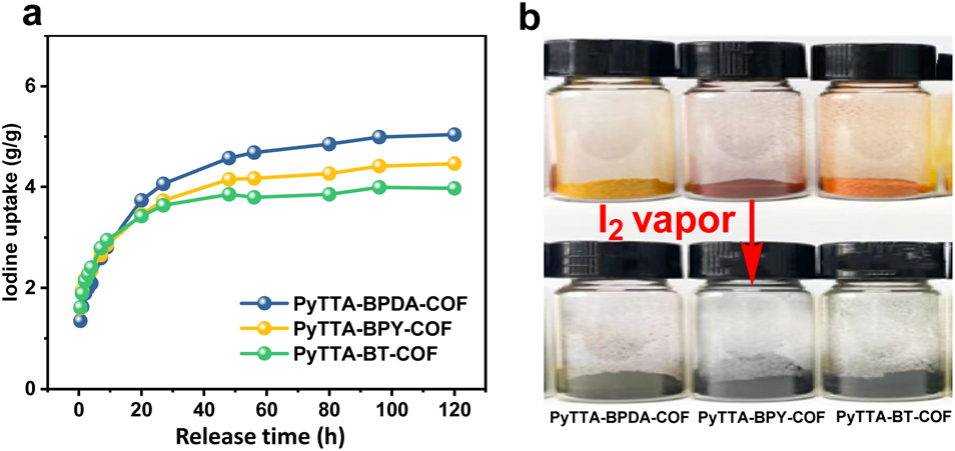
Iodine adsorption behavior of PyTTA-BPDA-COF, PyTTA-BPY-COF, and PyTTA-BT-COF (a) and their color changes (b).

Based on high BET surface areas and large pore volume as well as extended π-conjugation structure of the three COFs, we investigated iodine vapor adsorption within 120 hours at 75 °C and atmospheric pressure (the typical fuel reprocessing conditions). The experimental data (Fig. 4a) show that the adsorption of I_2_ significantly increased within the first 20 hours, followed by a slow rise. The adsorption process continued for 120 hours, and the sample weight remained almost constant, indicating the reach of adsorption equilibrium. The colors of the three COFs changed from red, brown, and orange-red to black, forming I_2_-loaded PyTTA-BPDA-COF (I_2_@PyTTA-BPDA-COF), PyTTA-BPY-COF (I_2_@PyTTA-BPY-COF), and PyTTA-BT-COF (I_2_@PyTTA-BT-COF), respectively (Fig. 4b). The corresponding iodine adsorption capacities reach 5.03, 4.46, and 3.97 g g^−1^, respectively. The iodine adsorption capacity of the three materials is comparable with those of reported adsorbent materials, as listed in Table S2.† Among the three, the iodine adsorption capacity of PyTTA-BPDA-COF is higher than those of the other two. The imine group is one of the important adsorption sites, and the density of imine group in PyTTA-BPDA-COF and PyTTA-BPY-COF is higher than PyTTA-BT-COF (Table S1†).

Therefore, PyTTA-BT-COF exhibited the lowest adsorption capability. Although PyTTA-BPDA-COF and PyTTA-BPY-COF have a similar imine density, the introduction of electronegative N in BPY decreased the basicity of imine, which led to the decrease in iodine adsorption capability. As a result, the adsorption of PyTTA-BPDA-COF is slightly higher than PyTTA-BPY-COF.

### Iodine release behavior and recyclability

**Fig. 5:**
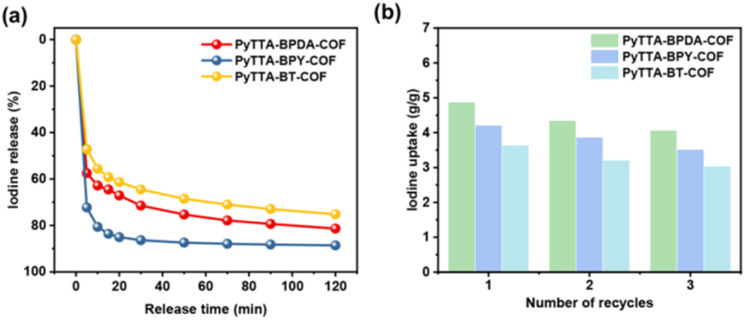
Release behavior as a function of time (a) cyclic adsorption capacities (b) of PyTTA-BPDA-COF, PyTTA-BPY-COF, and PyTTA-BT-COF.

Firstly, the UV-vis spectra of the three iodine-loaded materials in methanol solution at different times were identified (Fig. S1†). All three iodine-loaded materials exhibited rapid release behavior of iodine within 120 min, especially in the first 20 min. It may be attributed to several factors: (1) solvent effect: methanol, as a polar solvent, may interact with iodine molecules or the COF surface, weakening the binding between iodine and COFs to lead to rapid iodine release; (2) diffusion effect: due to the porous structure of COFs, methanol molecules contact with the loaded iodine molecules, promoting the diffusion and release of iodine molecules from the COF channels; (3) desorption effect: in the methanol solution, iodine-loaded COFs may undergo desorption. With the continuous penetration of methanol molecules and their interaction with iodine molecules, iodine molecules may gradually desorb from the COF surface and release into the solution; (4) COF-methanol interaction: the interaction with methanol may alter the structure or properties of COFs, thereby affecting the adsorption stability of iodine. It may be conducive for iodine molecules to be released from COFs. Additionally, through calculation, the desorption rates of the three iodine-loaded materials are 81%, 88%, and 75%, respectively (Fig. 5a). To verify the cyclic performance of iodine adsorption by PyTTA-BPDA-COF, PyTTA-BPY-COF, and PyTTA-BT-COF, iodine-loaded materials were subjected to Soxhlet extraction for iodine desorption. Then, the materials were reused for iodine adsorption. After three cycles, the iodine adsorption capacities of PyTTA-BPDA-COF, PyTTA-BPY-COF, and PyTTA-BT-COF remained at 4.02, 3.61, and 3.43 g g^−1^, respectively (Fig. 5b). This indicates that the iodine adsorption process is partially reversible due to the strong interaction between COFs and the adsorbed iodine.^27^

### Investigation of adsorption mechanism

**Fig. 6:**
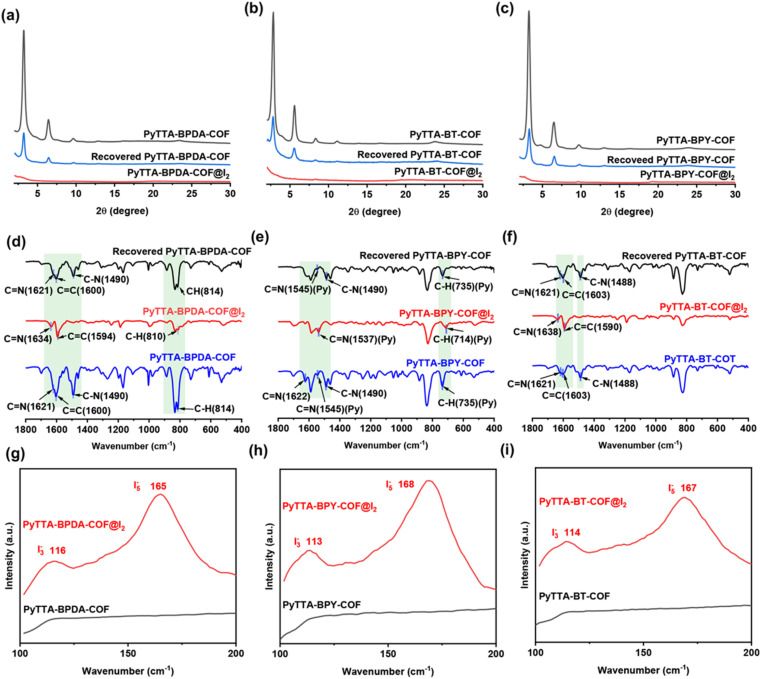
PXRD spectra of PyTTA-BPDA-COF (a), PyTTA-BPY-COF (b), PyTTA-BT-COF (c) before and after I_2_ adsorption and desorption. FT-IR spectra spectroscopy of PyTTA-BPDA-COF (d), PyTTA-BPY-COF (e), PyTTA-BT-COF (f) before and after I_2_ adsorption and desorption. Raman spectra of PyTTA-BPDA-COF (g), PyTTA-BPY-COF (h), PyTTA-BT-COF (i) before and after I_2_ adsorption and desorption.

We conducted PXRD, FT-IR and Raman tests on the COFs before and after iodine loading to analyze the iodine adsorption mechanism. The PXRD analysis was conducted on the materials after iodine adsorption (Fig. 6a–c). The results imply the disappearance of crystallinity after iodine loading, possibly due to the following two reasons: (1) interplanar spacing alternation: after iodine adsorption, the interplanar spacing of COFs may be significantly changed. The adsorption of iodine molecules into the COF pores or onto the surface may lead to adjustments in the crystal structure of COFs (an increase or decrease in the original interplanar spacing), thereby affecting the peak positions in the XRD pattern; (2) amorphous structure: upon iodine adsorption, COFs may partially or completely transform into an amorphous structure. This transformation may be attributed to the strong interaction between iodine and COFs, disrupting the original crystal structure. Additionally, we also tested the iodine-loaded materials after methanol desorption and found the recovery of crystal diffraction peaks. However, the diffraction peaks are somewhat lower compared to the original materials. The reason may be the strong interaction between iodine molecules and COFs prevents complete desorption.

The FT-IR spectra during iodine adsorption were illustrated in Fig. 6d–f. The blue shift occurs in the C=N bonds of the three COF materials, which may be attributed to the following four reasons: (1) changes in electron cloud distribution: the interaction between iodine molecules and the imine bonds in COFs may change the distribution of electron clouds around the imine bonds, potentially abating the electron density of the imine bonds, promoting the vibration frequency of the bonds, and causing the absorption spectrum to shift toward shorter wavelengths, *i.e.*, the blue shift; (2) polarization effect: due to its strong electronegativity, iodine may induce polarization when interacting with the imine bonds in COFs, intensifying the uneven distribution of electron clouds around the imine bonds, further affecting the vibration frequency of the bonds, and resulting in the blue shift; (3) conjugation effect: the imine bonds in COFs typically participate in conjugated systems, and the adsorption of iodine molecules may alter the electron distribution of the conjugated systems. This conjugation effect may impact the energy levels of the imine bonds, thereby causing the blue shift in the absorption spectrum; (4) intermolecular interactions: the interactions between iodine molecules and COFs may include van der Waals forces and hydrogen bonding, modifying the vibration modes and frequencies of the imine bonds and leading to the blue shift in the absorption spectrum. Additionally, it demonstrates that during adsorption, the reaction between the C=N bond and iodine formed polyiodide ions. Notably, after desorption, the chemical shift of the C=N bond returned to its original level, proving the reversibility and reusability of COFs.^28^

To delve into the forms of iodine adsorbed by the three COFs, Raman spectra before and after iodine adsorption were identified (Fig. 6g–i). The data results show no significant peaks in the Raman spectra of these COFs before iodine adsorption. After iodine capture, two characteristic peaks were observed at 116 and 165 cm^−1^ for PyTTA-BPDA-COF, at 113 and 168 cm^−1^ for PyTTA-BPY-COF, and at167 cm^−1^ for PyTTA-BT-COF. These peaks can be attributed to: (1) formation of polyiodide anions: when iodine is adsorbed into COFs, it may exist in the form of polyanion, such as I_3_^−^ and I_5_^−^. These polyanions have specific vibrational modes, which manifest as characteristic peaks in the Raman spectra; (2) symmetric and asymmetric stretching vibrations: the two characteristic peaks in the Raman spectra typically correspond to the symmetric and asymmetric stretching vibrations of I_3_^−^ and I_5_^−^. These vibrations arise from the positional variation of iodine atoms in the anion structure;^29^ (3) charge transfer and interaction: iodine adsorption results in charge transfer from COFs to iodine, further affecting the vibrational states of iodine. This charge transfer, along with the interaction between iodine and COFs, contributes to the appearance of characteristic peaks in the Raman spectra; (4) COF structure: the specific structures of COFs, such as the cage-like pattern, may provide distinct binding sites for iodine adsorption. These sites may influence the vibrational modes of iodine, resulting in specific characteristic peaks in the Raman spectra. Consequently, strong interactions exist between the constructed COFs and the adsorbed iodine.^30,31^ The generation of polyiodide anions was attributed to the formation of CT complexes between the electron-donating nitrogen adsorption binding sites and iodine molecules.^32^

## Conclusions

In summary, three extended π-conjugation pyrene-based two-dimensional (2D) covalent organic frameworks (COFs), namely, PyTTA-BPDA-COF, PyTTA-BPY-COF, and PyTTA-BT-COF have been successfully synthesized. These three COFs exhibit satisfying crystallinity and high specific surface area. The synergistic effect of the π-conjugated system in the nanochannels, and the imine bonds effectively enhances the adsorption/desorption capacity for iodine vapor. Experimental results demonstrate the superior adsorption performance of these three COFs for iodine vapor, alongside a notable release rate of iodine-loaded COFs in methanol solution. Testing methods such as infrared spectrum and Raman spectrum further reveal the mechanism of the materials adsorbing iodine, namely, the π-conjugated system and electron-rich sites collectively promote the formation of charge-transfer complexes between the adsorbent materials and iodine. Moreover, it underscores the significant enhancement in iodine adsorption capacity due to π-conjugated structure.

## Data availability

The data supporting this article have been included as part of the ESI.†

## Author contributions

W. H. performed the preparation and characterizations, including NMR, PXRD, and the uptake of iodine. H. H. analyzed the crystal structure. Others assisted with the experiments. J. Y. and S. X. analyzed the data and wrote the paper. Y. G. supervised the project.

## Conflicts of interest

There are no conflicts to declare.

## Supplementary Material

RA-014-D4RA04994B-s001

## References

[cit1] Riley B. J., Vienna J. D., Strachan D. M., McCloy J. S., Jerden J. L. (2016). J. Nucl. Mater..

[cit2] Huve J., Ryzhikov A., Nouali H., Lalia V., Augé G., Daou T. J. (2018). RSC Adv..

[cit3] Xie W., Cui D., Zhang S., Xu Y., Jiang D. (2019). Mater. Horiz..

[cit4] Wang X., Zeng W. L., Song M. J., Wang F. L., Hu X. D., Guo Q. J., Liu Y. Z. (2019). Chem. Eng. J..

[cit5] Wang X., Guo Q. J., Kong T. T. (2015). Chem. Eng. J..

[cit6] Wang K. L., Zhou T. T., Cao Z., Yuan Z. M., He H. Y., Fan M. H., Jiang Z. Y. (2024). Green Energy Environ..

[cit7] Chen H., Gao Y., Li J., Fang Z., Bolan N., Bhatnagar A., Gao B., Hou D., Wang S., Song H., Yang X., Shaheen S. M., Meng J., Chen W., Rinklebe J., Wang H. (2022). Carbon Res..

[cit8] Subrahmanyam K. S., Malliakas C. D., Sarma D., Armatas G. S., Wu J., Kanatzidis M. G. (2015). J. Am. Chem. Soc..

[cit9] Elmore D., Gove H. E., Ferraro R., Kilius L. R., Lee H. W., Chang K. H., Beukens R. P., Litherland A. E., Russo C. J., Purser K. H., Murrell M. T., Finkel R. C. (1980). Nature.

[cit10] Jin K., Lee B., Park J. (2021). Coord. Chem. Rev..

[cit11] Li J., Jing X., Li Q., Li S., Gao X., Feng X., Wang B. (2020). Chem. Soc. Rev..

[cit12] Geng K., He T., Liu R., Dalapati S., Tan K. T., Li Z., Tao S., Gong Y., Jiang Q., Jiang D. (2020). Chem. Rev..

[cit13] Zhang Z., Dong X., Yin J., Li Z.-G., Li X., Zhang D., Pan T., Lei Q., Liu X., Xie Y., Shui F., Li J., Yi M., Yuan J., You Z., Zhang L., Chang J., Zhang H., Li W., Fang Q., Li B., Bu X.-H., Han Y. (2022). J. Am. Chem. Soc..

[cit14] Zhu G. , Liu J., Liu J. and Luo R., in Dan Shen (Salvia Miltiorrhiza) in Medicine: Volume 3. Clinical Research, ed. X. Yan, Springer Netherlands, Dordrecht, 2015, pp. 153–202, 10.1007/978-94-017-9466-4_11

[cit15] Subrahmanyam K. S., Sarma D., Malliakas C. D., Polychronopoulou K., Riley B. J., Pierce D. A., Chun J., Kanatzidis M. G. (2015). Chem. Mater..

[cit16] Sava D. F., Rodriguez M. A., Chapman K. W., Chupas P. J., Greathouse J. A., Crozier P. S., Nenoff T. M. (2011). J. Am. Chem. Soc..

[cit17] Ma B., Zhou Y., Hu W., Zhang Y. (2024). New J. Chem..

[cit18] Li H., Zhang D., Cheng K., Li Z., Li P.-Z. (2023). ACS Appl. Nano Mater..

[cit19] Zhao Y., Lu W., Zhang Y., Liu X., Sun B. (2024). Microporous Mesoporous Mater..

[cit20] Khosravi Esmaeiltarkhani F., Dinari M., Mokhtari N. (2024). New J. Chem..

[cit21] Liu C., Xia M., Zhang M., Yuan K., Hu F., Yu G., Jian X. (2020). Polymer.

[cit22] Leng W., Peng Y., Zhang J., Lu H., Feng X., Ge R., Dong B., Wang B., Hu X., Gao Y. (2016). Chem.–Eur. J..

[cit23] Li Y., Yang L., He H., Sun L., Wang H., Fang X., Zhao Y., Zheng D., Qi Y., Li Z., Deng W. (2022). Nat. Commun..

[cit24] Li Z., Zhi Y., Shao P., Xia H., Li G., Feng X., Chen X., Shi Z., Liu X. (2019). Appl. Catal., B.

[cit25] Zhu Q., Wang X., Clowes R., Cui P., Chen L., Little M. A., Cooper A. I. (2020). J. Am. Chem. Soc..

[cit26] Hernández M. A., Rojas F., Lara V. H. (2000). J. Porous Mater..

[cit27] Cheng K., Li H., Li Z., Li P.-Z., Zhao Y. (2023). ACS Mater. Lett..

[cit28] Yang J., Wang S., Yan Q., Hu H., Xu H., Ma H., Su X., Gao Y. (2024). Polym. Chem..

[cit29] Cambedouzou J., Sauvajol J. L., Rahmani A., Flahaut E., Peigney A., Laurent C. (2004). Phys. Rev. B.

[cit30] Guo X., Li Y., Zhang M., Cao K., Tian Y., Qi Y., Li S., Li K., Yu X., Ma L. (2020). Angew. Chem., Int. Ed..

[cit31] Chang J., Li H., Zhao J., Guan X., Li C., Yu G., Valtchev V., Yan Y., Qiu S., Fang Q. (2021). Chem. Sci..

[cit32] Hsu S. L., Signorelli A. J., Pez G. P., Baughman R. H. (2008). J. Chem. Phys..

