# Supplementary material for: Exploration of iodine adsorption performance of pyrene-based two-dimensional covalent organic frameworks
Source: RSC Adv. 2024 Aug 15;14(35):25695–702. doi: 10.1039/d4ra04994b (PMC11325339; doi:10.1039/d4ra04994b)
Supplement: RA-014-D4RA04994B-s001 [file RA-014-D4RA04994B-s001.pdf]

Supplementary Information (SI) for RSC Advances.
This journal is © The Royal Society of Chemistry 2024

Exploration of Iodine Adsorption Performance of Covalent Organic Frameworks Enriched with Diverse Heteroatoms

Weican He,[a] Shenglin Wang,[a] Hui Hu,[a] Jiaxin Yang,*[a] Tiao Huang,[a] Xiaofang Su,[a] Songtao Xiao,*[b] Jianyi Wang,[a] and Yanan Gao*[a]

[a] Key Laboratory of Ministry of Education for Advanced Materials in Tropical Island Resources, Hainan University, No 58, Renmin Avenue, Haikou 570228, China.

[b] China Institute of Aromatic Energy, Beijing 102413, China

Characterization

Solution ^{1}H and ^{13}C NMR spectra were recorded on a Bruker 400 MHz NMR spectrophotometer. Solid-state ^{13}C CM/MAS NMR spectra were measured on a Bruker AVANCE III 600 MHz spectrometer. The electronic absorption spectra were recorded on a JASCO model V-770 spectrophotometer. Fourier-transform infrared (FT-IR) spectra were detected on a Jasco model FT/IR-6800 infrared spectrophotometer with a scan number of 32, and the background was subtracted. Powder X-ray diffraction (PXRD) data were performed on a Rigaku Smart Lab X-ray diffractometer with Cu K_α radiation (λ = 1.540598 Å) by depositing powder on glass substrate to measure at 2θ from 2 to 30° with a 0.05° increment. N_2 and CO_2 adsorption analyses were performed by using QUANTACHROME AUTOSORB-IQ2 to analyze the specific surface area, pore size distributions. Before measurement, powder samples were degassed under a dynamic vacuum at 120 °C for 15 h. Brunauer Emmett-Teller (BET) surface areas were calculated from the linear region of the N_2 isotherm at 77 K within the pressure range P/P_0 of 0.003–0.05 using micropore BET assistant on the ASiQwin software. Pore size distributions were determined using the quenched solid density functional theory (QSDFT) method. Thermogravimetric analysis (TGA) was recorded on a mettler TG-DSC 3+ under N_2 at a heating rate of 10 °C min^{-1} from ambient temperature to 800 °C. Elemental analysis (EA) of C, H and N was collected by Vario EL cube (Elementar, Germany) elemental analyzer.

Field emission scanning electron microscopy (FE SEM) was measured on the scanning electron microscopy (Gemini300, ZEISS Germany) at 3.0 kV acceleration voltage. High-resolution transmission electron microscope (HR-TEM) analysis was recorded on FEI Tecnai G2 F30 electron microscope. The Raman spectra were obtained using an inVia Qontor Evolution Raman spectrometer with a 532 nm laser.

Iodine Vapor Adsorption

The iodine vapor adsorption experiment was conducted following the literature [1]: Approximately 10 mg of COFs was placed in a glass vial A, and an excess of radioactive iodine was placed in another glass vial B. Then, vials A and B were placed together in a sealed container to expose the adsorbent to massive non-radioactive iodine. The sealed container was placed in an oven and heated at 75 °C. At regular intervals, vial A was taken out to measure its mass after cooling. The iodine adsorption capacity of the COF samples was calculated using Formula (2.1).

$$\alpha = \frac{m_t - m_0}{m_0} \quad (2.1)$$

Where α (g g^{-1}) represents the capacity of iodine adsorption, and m_0 and m_t denote the masses of the COF samples at the initial and t-th time points, respectively.

Desorption Experiment of Iodine-loaded COF Materials

Separately, 0.2 mg of PyTTA-BPDA-COF@I_2, PyTTA-BPY-COF@I_2, and PyTTA-BT-COF@I_2 were placed in nine sample bottles, adding 3 mL of methanol solution. After 5 min, 10 min, 15 min, 20 min, 30 min, 50 min, 70 min, 90 min, and 120 min, the liquid was filtered and subjected to UV-vis spectrophotometry, respectively. The iodine content in the solution was determined based on the standard curve, and the desorption rate was calculated using Formula (2.2).

$$w = \frac{(1 + C)\ m_d}{0.2C} * 100\% \ (2.2)$$

where w denotes the desorption rate, C (g/g) represents the adsorption quantity, and

m_d (g) signifies the mass of desorbed iodine.

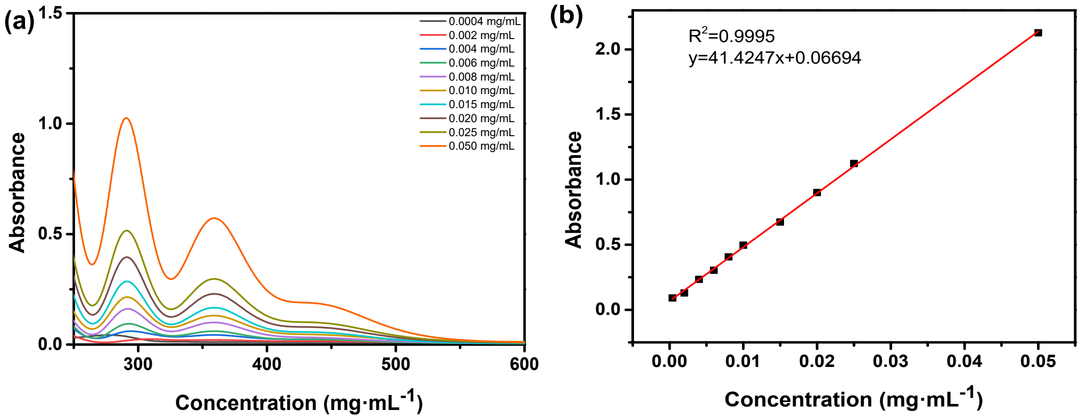

Fig. S1. UV-vis spectral of methanol standard solutions of iodine with different concentrations (a) and the corresponding calibration curve of absorbance versus iodine concentration (b) established from UV-vis spectra as shown in (a).

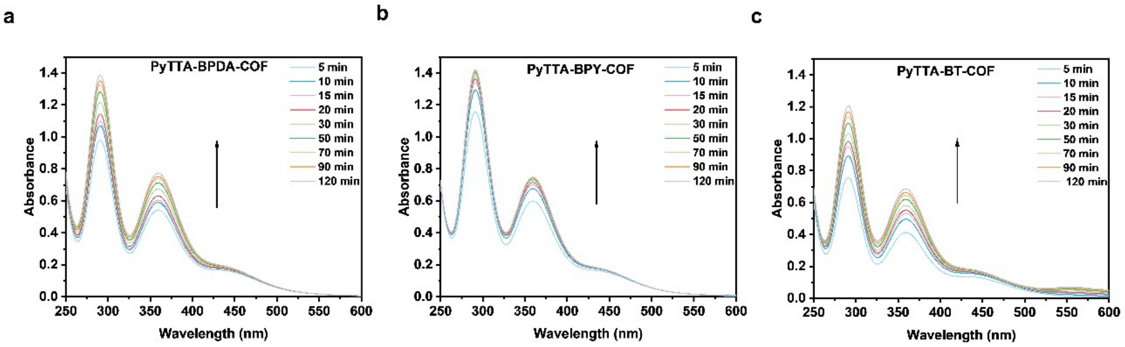

Figure S2. The UV-vis spectra of iodine desorption of I_2@PyTTA-BPDA-COF (a), I_2@PyTTA-BPY-COF (b), and I_2@PyTTA-BT-COF (c).

Table 1. Textural properties of 4F-Py-COF, IM4F-Py-COF, and BMIM4F-Py-COF.

COFs	S_{BET} (m^2 g^{-1})	Pore width (nm)	Total pore volume (cm^3 g^{-1})	Adsorption Capability (g g^{-1})	C=N density
PyTTA-BPDA-COF	869.13	2.5	0.78	5.03	11.4%
PyTTA-BPY-COF	1136.19	2.6	0.94	4.46	11.3%
PyTTA-BT-COF	707.99	2.9	0.57	3.97	8.8%

Table S2. Comparison of representatively reported adsorbents with our work for iodine vapor adsorption under atmospheric pressure.

Sample	BET (m^2 g^{-1})	Capture of iodine vapor (g g^{-1})	Ads. temp	Ads. time	Ref.
COF-TAPT	2348	8.61	75 °C	96 h	[2]
COF-TAPB	2290	7.94	75 °C	96 h	[2]
TFB-DB COF	-	6.40	75 °C	72 h	[3]
TFB-BD COF	-	6.23	75 °C	72 h	[3]
QTD-COF-V	-	6.29	75 °C	4 h	[3]
TPB-DMTP COF	1927	6.26	75 °C	96 h	[4]
TJNU-203	1833	5.88	77 °C	120 h	[5]
TJNU-201	2510	5.62	75 °C	96 h	[6]
TPT-BD COF	109	5.43	75 °C	46 h	[3]
TTDP-1	12	5.3	75 °C	22 h	[7]
CMP-LS8	2028	5.29	75 °C	12 h	[8]
QTD-COF-3	-	5.16	75 °C	6 h	[3]
Car-CMP-3	450	5.10	75 °C	120 h	[9]
**PyTTA-BPDA-COF**	**869**	**5.03**	**75 °C**	**120 h**	**This work**

CMPN	86.2	5.02	60 °C	140 h	10
Car-CMP-1	305	4.98	75 °C	120 h	9
TTA-TTB COF	1733	4.95	75 °C	96 h	4
DbTd-COF	368	4.93	75 °C	48 h	11
CSUCPOP-1	1032	4.9	75 °C	30 h	12
QTD-COF-4	-	4.85	75 °C	4 h	3
TJNU-202	714	4.82	75 °C	96 h	6
ETTA-TPA	1822	4.79	75 °C	96 h	4
TTDP-2	7	4.7	75 °C	22 h	7
TPE-PyTTA-CMP	-	4.68	75 °C	120 h	13
QTD-COF-1	-	4.67	75 °C	4 h	3
TPT-DHBD25 COF	188	4.65	75 °C	46 h	3
DaTd-COF	275	4.48	75 °C	48 h	11
**PyTTA-BPY-COF**	**1136**	**4.46**	**75 °C**	**120 h**	**This work**
CMP-LS5	-	4.4	75 °C	110 h	14
TPT-DHBD50 COF	124	4.30	75 °C	46 h	3
TTDP-3	13	4.2	75 °C	22 h	7
i-POP-BPTM-3	1485	4.15	77 °C	24 h	15
TPT-DHBD75 COF	157	4.12	75 °C	46 h	3
CSUCPOP-2	555	4.1	75 °C	30 h	12
P-DPDA	24	4.08	75 °C	8 h	16
TPT-DHBD COF	297	4.03	75 °C	46 h	3
**PyTTA-BT-COF**	**707**	**3.97**	**75 °C**	**120 h**	**This work**
i-POP-BPTM-2	1611	3.75	77 °C	24 h	15
TPE-TAPP-CMP	-	3.67	75 °C	120 h	13
DpTd-COF	127	3.43	75 °C	48 h	11
i-POP-BPTM-1	1753	3.42	77 °C	24 h	15
Car-CMP-2	412	3.38	75 °C	120 h	9
P-TPB	646	3.35	75 °C	8 h	16
CMP-LS4	-	3.32	75 °C	110 h	14

CSUCPOP-3	269	3.3	75 °C	30 h	12
Bpy-Cage	1.8	3.23	75 °C	24 h	17
BTPOC	52	3.21	75 °C	50 h	18
HCMP3	82	3.16	75 °C	110 h	19
PTPATTh	-	3.13	75 °C	60 h	20
TPE-TPDA-CMP	-	3.1	75 °C	120 h	13
QTD-COF-2	-	2.87	75 °C	5 h	3
COF-TpgDB	209	2.75	125 °C	72 h	21
PTPATCz	-	2.56	75 °C	60 h	20
CMP-LS6	-	2.44	75 °C	110 h	14
ImCMP-1	-	2.36	80 °C	8 h	22
COF-TpgBD	217	1.81	125 °C	72 h	21
COF-TpgTd	303	1.66	125 °C	72 h	21

References:

1. Y. B. Zhang, H. Furukawa, N. Ko, W. Nie, H. J. Park, S. Okajima, K. E. Cordova, H. Deng, J. Kim and O. M. Yaghi, *J. Am. Chem. Soc.*, 2015, **137**, 2641-2650.
2. Y. Xie, T. Pan, Q. Lei, C. Chen, X. Dong, Y. Yuan, W. A. Maksoud, L. Zhao, L. Cavallo, I. Pinnau and Y. Han, *Nature Communications*, 2022, **13**, 2878.
3. X. Guo, Y. Li, M. Zhang, K. Cao, Y. Tian, Y. Qi, S. Li, K. Li, X. Yu and L. Ma, *Angewandte Chemie International Edition*, 2020, **59**, 22697-22705.
4. P. Wang, Q. Xu, Z. Li, W. Jiang, Q. Jiang and D. Jiang, *Advanced Materials*, 2018, **30**, 1801991.
5. L. Zhang, J. Li, H. Zhang, Y. Liu, Y. Cui, F. Jin, K. Wang, G. Liu, Y. Zhao and Y. Zeng, *Chemical Communications*, 2021, **57**, 5558-5561.
6. X. Guo, Y. Tian, M. Zhang, Y. Li, R. Wen, X. Li, X. Li, Y. Xue, L. Ma, C. Xia and S. Li, *Chemistry of Materials*, 2018, **30**, 2299-2308.
7. W. Du, Y. Qin, C. Ni, W. Dai and J. Zou, *ACS Applied Polymer Materials*, 2020, **2**, 5121-5128.
8. S. Wang, Q. Hu, Y. Liu, X. Meng, Y. Ye, X. Liu, X. Song and Z. Liang, *Journal of Hazardous Materials*, 2020, **387**, 121949.
9. J. Yang, S. Wang, Q. Yan, H. Hu, H. Xu, H. Ma, X. Su and Y. Gao, *Polymer Chemistry*, 2024, DOI: 10.1039/D4PY00425F.
10. M. Xu, T. Wang, L. Zhou and D. Hua, *Journal of Materials Chemistry A*,

2020, **8**, 1966-1974.
11. Z. Wu, W. Wei, J. Ma, J. Luo, Y. Zhou, Z. Zhou and S. Liu, *ChemistrySelect*, 2021, **6**, 10141-10148.
12. S. Xiong, X. Tang, C. Pan, L. Li, J. Tang and G. Yu, *ACS Applied Materials & Interfaces*, 2019, **11**, 27335-27342.
13. S. Luo, Q. Yan, S. Wang, H. Hu, S. Xiao, X. Su, H. Xu and Y. Gao, *ACS Applied Materials & Interfaces*, 2023, **15**, 46408-46416.
14. S. Wang, Y. Liu, Y. Ye, X. Meng, J. Du, X. Song and Z. Liang, *Polymer Chemistry*, 2019, **10**, 2608-2615.
15. Z. Li, H. Li, D. Wang, A. Suwansoontorn, G. Du, Z. Liu, M. M. Hasan and Y. Nagao, *Polymer*, 2020, **204**, 122796.
16. J. Wang, C. Wang, H. Wang, B. Jin, P. Zhang, L. Li and S. Miao, *Microporous and Mesoporous Materials*, 2021, **310**, 110596.
17. D. Luo, Y. He, J. Tian, J. L. Sessler and X. Chi, *Journal of the American Chemical Society*, 2022, **144**, 113-117.
18. C. Liu, Y. Jin, Z. Yu, L. Gong, H. Wang, B. Yu, W. Zhang and J. Jiang, *Journal of the American Chemical Society*, 2022, **144**, 12390-12399.
19. Y. Liao, J. Weber, B. M. Mills, Z. Ren and C. F. J. Faul, *Macromolecules*, 2016, **49**, 6322-6333.
20. T. Geng, G. Chen, H. Xia, W. Zhang, Z. Zhu and B. Cheng, *Journal of Solid State Chemistry*, 2018, **265**, 85-91.
21. Y. Sun, S. Song, D. Xiao, L. Gan and Y. Wang, *ACS Omega*, 2020, **5**, 24262-24271.
22. X. Meng, Y. Liu, S. Wang, Y. Ye, X. Song and Z. Liang, *Microporous and Mesoporous Materials*, 2022, **336**, 111871.
